# Genetic variability in geographic populations of the natterjack toad (*Bufo calamita*)

**DOI:** 10.1002/ece3.323

**Published:** 2012-07-20

**Authors:** N Oromi, A Richter-Boix, D Sanuy, J Fibla

**Affiliations:** 1 Escola Tècnica Superior d'Enginyeria Agrària, Departament of Producció Animal (Fauna Silvestre), University of Lleida25198, Lleida, Spain; 2 Campus de Ciències de la Salut, Edifici Biomedicina I, Department of Basic Medical Sciences, Universitat de Lleida-IRBLleida25198, Lleida, Spain; 3 Department of Ecology and Evolution, Evolutionary Biology Centre, Uppsala University75236, Uppsala, Sweden

**Keywords:** Altitudinal and latitudinal gradient, *Bufo calamita*, genetic diversity, microsatellite markers

## Abstract

Across altitudinal and latitudinal gradients, the proportion of suitable habitats varies, influencing the individual dispersal that ultimately can produce differentiation among populations. The natterjack toad (*Bufo calamita)* is distributed across a wide geographic range that qualifies the species as interesting for a geographic analysis of its genetic variability. Five populations of *B. calamita* in the Sierra de Gredos (Spain) were studied in an altitudinal gradient ranging from 750 to 2270 m using microsatellite markers. In addition, we analyzed the latitudinal genetic variation in *B. calamita* within a global European distribution using genetic diversity parameters (mean number of alleles per locus [*M*_*a*_] and expected heterozygosity [*H*_*E*_]) obtained from our results and those published in the literature. The low level of genetic differentiation found between populations of *B. calamita* (*F*_*st*_ ranging from 0.0115 to 0.1018) and the decreases in genetic diversity with altitude (*M*_*a*_ from 13.6 to 8.3, *H*_*E*_ from 0.82 to 0.74) can be interpreted by the combined effects of discontinuous habitat, produced mainly by the high slopes barriers and geographic distance. In the latitudinal gradient, genetic diversity decreases from south to north as a consequence of the colonization of the species from the Pleistocene refugium. We conclude that the genetic variability in *B. calamita* along its wide altitudinal and latitudinal geographic distribution mainly reflects the colonization history of the species after the last glacial period.

## Introduction

Genetic variation is required for the evolution of populations in response to environmental changes (Reed and Frankham 2003). Environmental factors such as altitude, topography, and glacial history may influence genetic variation. Across altitudinal and latitudinal gradients, the proportion of suitable habitats varies, influencing the individual dispersal that ultimately can produce differentiation among populations (Palo et al. 2003; Stéphanie et al. 2003). In fact, different selective pressures acting on local environments across latitudinal and altitudinal gradients added to a subjacent genetic diversity may derive from a local adaptation (Slatkin 1987; Palo et al. 2003; Bonin 2006; Rogell et al. 2009). However, the historical events experienced by a population can also drive local differentiation. In species with a wide geographic distribution and a glacial colonization history, it can be difficult to discriminate between genetic diversity that results from postglacial colonization patterns and genetic differentiation that results from habitat influences (e.g., local selective pressures, recent habitat fragmentation; Allentoft et al. 2009). As these two processes act at very different spatial and temporal scales, they can have different effects on genetic diversity and fitness (Swindell and Bouzat 2006)

In amphibians, metapopulation structures generally have a high gene flow that can preclude complete differentiation between populations over large geographic distances (Brede and Beebee 2004). In fact, the migratory range of species determines its capacity to maintain the genetic cohesion within local populations that favors the persistence of the species in its distribution range (Smith and Green 2005). Thus, the dispersal range, the population size, and the genetic relationships between individuals are essential to understanding the evolution of a species (Petit et al. 2001). In this sense, the study of genetic variability is important to determine the levels of genetic differentiation among populations at both geographic and altitudinal distance scales. Genetic differentiation between populations was positively correlated with geographic distance in several amphibian studies (e.g., in *Rana temporaria*, Palo et al. 2004; in *Pelophylax esculentus*, Arioli et al. 2010) at a large scale. The impact of altitudinal gradient on dispersal and gene flow seems to differ between species. For example, genetic variation was negatively correlated with altitude in the frog *Rana luteiventris* (Funk et al. 2005) and in the salamander *Ambystoma macrodactulym* (Giordano et al. 2007), whereas no correlation was detected in *Rana chensinensis* (Zhan et al. 2009). Genetic differentiation by geographic (isolation by distance) or altitudinal distance (a combination of isolation by distance and isolation by geographic barriers) evolves over time and arises from the balance of local genetic drift within populations and dispersal of individuals between populations.

The natterjack toad (*Bufo calamita)* is distributed across a wide geographic range (Sinsch 2008) that qualifies the species as interesting for a geographic analysis of its genetic variability. Previous studies of geographic genetic differentiation using polymorphic microsatellite loci in *B. calamita* in lowland populations (at 0–400 m) of the southern Iberian Peninsula found no genetic differentiation between populations separated by more than 100 km (Marangoni 2006). Little population differentiation and a lack of isolation by distance pattern were also found in populations in several breeding sites with different salinity levels in southern Spain (Gomez-Mestre and Tejedo 2004). However, species distribution studies covering a broad latitudinal range show a negative correlation between genetic variation and distance from the Iberian Peninsula, which is the Pleistocene glacial refuge from which all extant populations are derived (Beebee and Rowe 2000; Rowe et al. 2006). In contrast, the altitudinal effect in *B. calamita* genetic variability has not yet been assessed.

This study analyses the impact of both altitude and geographic distance, in an effort to expand what is known about *B. calamita* genetic variability. We used expected heterozygosity and allelic richness as components of genetic diversity. Some authors consider that allelic richness is an important measure of genetic diversity and a relevance key in conservation programs (Petit et al. 1998; Simianer 2005; Foulley and Ollivier 2006). Allelic diversity is particularly important from a long-term perspective because the limit of selection response is mainly determined by the initial number of alleles regardless of the allelic frequencies (Hill and Rasbash 1986) and because it reflects better past fluctuations in population size. As the maximum altitudinal range of natterjacks distribution is in the mountains of the Iberian Peninsula (at 2400 m in the Sierra de Gredos and 2540 m in the Sierra Nevada; Sinsch 2008) we chose five populations inhabiting the Sierra de Gredos ranges from 750 to 2270 m. The study aimed to (i) characterize the genetic diversity of each population, (ii) analyze the genetic differences among populations in an altitudinal gradient, (iii) analyze whether mountains constitute natural barrier for *B. calamita* affecting genetic diversity among populations across an altitudinal gradient, and (iv) evaluate the consequences of mountains as barriers across European distribution, studying its latitudinal genetic variation using genetic diversity parameters obtained from published studies.

## Material and Methods

### Study sites and population sampling

**Figure 1:**
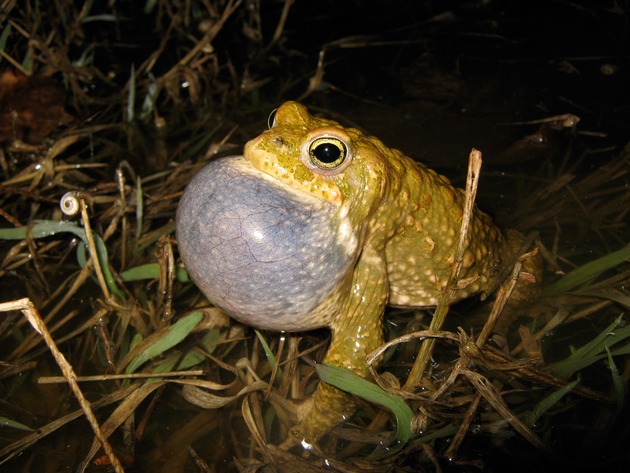
A detailed photography of a male individual of the natterjack toad *Bufo calamita,* in the studied zone of Sierra de Gredos (Spain).

**Figure 2:**
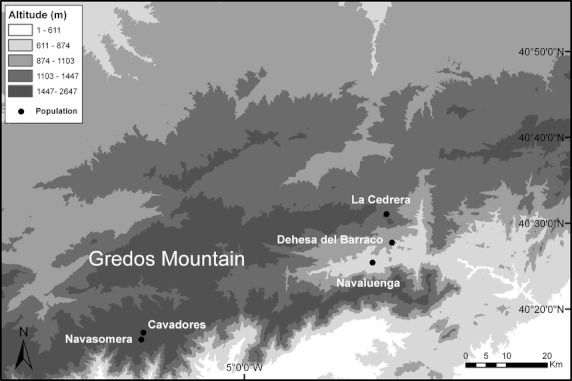
Gredos mountain map of elevation including the location of the five populations considered in this study.

A total of five populations of natterjack toads (*B. calamita*) (Fig. 1) were studied on the north side of the Sierra de Gredos (Central Iberian System, Spain), following the altitudinal gradient of the mountains: Navaluenga, Nav750; La Dehesa del Barraco, Deh920; La Cedrera, LaC1470; Cavadores, Cav2100; and Navasomera, Nas2300 (Supporting Table S1) (Fig. 2). The climate is Mediterranean with an average mean temperatures range from 6 to 12°C, with a range between 0 and 2°C during the coldest months (December, January, and February) and 20–22°C during the hottest months (July and August) depending on the altitude (Ninyerola et al. 2005). Precipitation ranges from 1000 to >2000 mm (Palacios et al. 2003). The breeding sites of *B. calamita* in the Sierra de Gredos are humid meadows and ponds at lower sites, and glacial lagoons at higher altitudes.

Toads were captured at the local breeding ponds during the spring reproduction period. They were released in situ after sex determination, snout-vent length (SVL) measurement, and toe-clipping (third toe of the right hind limb). The toes were stored in 70% ethanol at room temperate. The DNA was extracted from the first phalange of a toe clipped using the Chelex100 protocol described in (Walsh et al. 1991). The other two phalanges were used for skeletochronological analysis that had been the aim of another study (Oromi et al. 2012).

### Microsatellite analysis

Genetic analysis was based on the study of eight microsatellite loci previously described by Rowe et al. (1997) (*Bcalμ1***,** *Bcalμ2*, *Bcalμ3*, *Bcalμ4*, *Bcalμ5*, *Bcalμ6*, *Bcalμ7)* and Rowe et al. (2000) (*Bcalμ10*). Following the methodology used by (Gomez-Mestre and Tejedo 2004) genotypes at microsatellite polymorphism were determined by polymerase chain reaction (PCR) amplification using fluorescence labeled primers. Briefly, the oligo forward of each set of primers was stained by one of the four FAM, VIC, NED, and PET fluorochromes. PCR for each set of primers contained: 2.5 mmol/L MgCl_2_, 0.1 mmol/L BSA, 0.25 mmol/L dNTPs, Taq DNA polymerase 1 U, forward and reverse primers 0.25 *μ*mol/L each, and DNA 2 *μ*L (50–150 ng) in 20 *μ*L of PCR buffer 1×. The PCR amplification was performed by first denaturing at 94°C for 5 min, followed by 40 cycles of 1 min at 94°C, 1 min at 62°C, and 1 min at 72°C. A final extension step was carried out by incubating samples during 10 min at 72°C. Amplified products were resolved by capillary gel electrophoresis on a Genetic Analyser 3130 (Applied Biosystems, Foster City, CA) using POP-7 polymer. Allele sizes were determined using GeneScan-500 LIZ standard marker (ABI-PRISM, Applied Biosystems). Genotype calls were obtained using GeneMapper Software v. 4.0.

### Data analysis

Allele and genotype frequencies, number of alleles per locus, mean number of alleles per locus (*M*_*a*_), and expected (*H*_*E*_) and observed (*H*_*o*_) heterozygosity were estimated using the Microsatellite Toolkit (Park 2001). Allelic richness (*AR*) was obtained for each population using FSTAT v. 2.9.3 (Goudet 2001). *M*_*a*_, *AR*, and *H*_*E*_ were used as indicators of genetic diversity. Although *AR* reflects better past fluctuations than *M*_*a*_ (Hill and Rasbash 1986), both genetic diversity parameters were used in order to compare our results with those published in the literature in which *M*_*a*_ has been used as a measure of diversity. The influence of altitude on the *H*_*E*_, *AR*, and *M*_*a*_ was estimated by fitting data to several simple regression models. We chose the double reciprocal model for *H*_*E*_, S-curve model for *AR*, and reciprocal-X model for *M*_*a*_ predictions because they provided the best fit, defined as maximum *R*². The analysis was performed using the statistical package STATGRAPHICS Plus 5.0. Genotype frequencies were tested for conformity to Hardy–Weinberg equilibrium by GENEPOP v.3.4 (Raymond and Rousset 1995) using the Markov chain method with 10,000 permutations. This package was also used to estimate the fixation indices *F*_*st*_ and *R*_*st*_ and to evaluate marker-to-marker genotypic disequilibrium adjusting for Bonferroni correction. As all markers used are in different chromosomes (Rowe et al. 1997, 2000), the genotypic linkage equilibrium was tested as a measure of locus association that could inform us about population structure.

The analysis of genetic structure was made with an initial comparison within and among the three sampling regions (Altitudinal groups: (i) less than 1000 m: Nav750 and Deh920, (ii) between 1000 and 2000 m: LaC1470, and (iii) more than 2000 m: Cav2100 and Nas2300). The data were analyzed with a molecular analysis of variance (nested AMOVA) using ARLEQUIN v. 3.1 (Excoffier et al. 2006). The geographic and altitudinal pattern of genetic variation was analyzed using a partial Mantel test (10,000 permutations) carried out between genetic distance matrices (based on *F*_*st*_) and geographic and altitudinal distance matrices using the PASSAGE v.2. software (Rosenberg and Anderson 2011). A third constant matrix was used in both tests (geographic matrix for *F*_*st*_ and altitude correlation; altitude matrix for *F*_*st*_ and geographic correlation) for the accurate estimation of Mantel test statistics.

### Latitudinal genetic variation

In addition to data obtained in this study, *B. calamita* genetic diversity data were compiled from literature (Supporting Table S2). In total, 57 populations from different localities of natterjack toad distribution were included in the data analysis. The mean number of alleles per locus (*M*_*a*_*, n* = 34) and the expected heterozygosity (*H*_*E*_, *n* = 57) were used as estimators of genetic diversity. The influence of latitude on the *M*_*a*_ and on the *H*_*E*_ was estimated by fitting data in two-simple regression models: the lineal model and the exponential model, which represent two predefined hypothesis. The lineal model is expected when genetic diversity decrease constantly along the latitudinal gradient following an isolation by distance pattern. However, the exponential model is expected in the presence of barriers bursting the continuous decrease in genetic diversity from the glacial refuge across the latitudinal gradient. We also analyzed the data separately between the populations from Spain (glacial refuge) and those past the Pyrenees (post glacial colonization) in order to evaluate the expansion of the species. The analysis was performed using the statistical package STATGRAPHICS Plus 5.0.

## Results

### Population genetic diversity

The eight microsatellite markers used were polymorphic in all populations and the number of alleles per locus varied from 14 for *Bcalμ7* to 36 for *Bcalμ4*. We identified a total of 174 different alleles; 32 were present in all populations (common alleles) and 72 were observed in unique populations (private alleles). *H*_*E*_ ranged from 0.44 at Cav2100 to 0.93 at Nav750 (Supporting Table S3), very close to *H*_*o*_ values. Deviation from Hardy–Weinberg equilibrium was observed for *Bcalμ3* in the Cav2100 population, caused by heterozygote deficiency that could be explained by nonamplifying alleles. After Bonferroni correction for multiple tests, a significant deviation from genotypic linkage equilibrium was found in six of the total 139 locus pair tested (data not shown). The low deviation from linkage equilibrium observed could be interpreted as a relaxed population structure.

**Figure 3:**
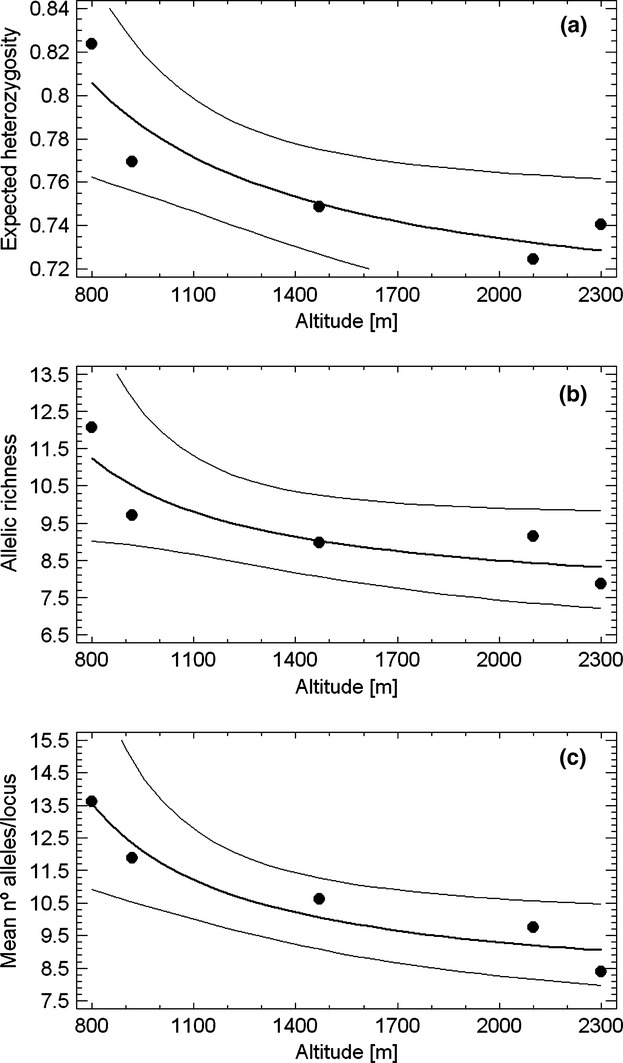
Genetic diversity and altitude relationship. Mean expected heterozygosity (a) and allelic richness (b) for each population. Each point represents a population sample. Confidence limit (95%) is shown in the figure.

Altitude correlated significantly and inversely with the parameters of genetic diversity, explaining the 85.02% of the variance in percentage of *H*_*E*_ (*H*_*E*_ *=* 1/[1.44276 + 161.275/Altitude]; *r* = *−*0.922, *P* = 0.026, Fig. 3a), the 77.01% of the variance in *AR* (*AR* = exp [1.95193 + 374.979/Altitude]; *r* = 0.877, *P* = 0.05; Fig. 3b), and the 92.55% of the variance in *M*_*a*_ (*M*_*a*_ = 6.70675 + 5278.81/Altitude; *r* = 0.96, *P* = 0.008; Fig. 3c).

Differences between populations, measured by *F*_*st*_ and *R*_*st*_ were low, ranging from 0.0115 to 0.1018 and 0.0062 to 0.1148, respectively. The partial Mantel test used to evaluate the correlation of *F*_*st*_ matrices with geographic and altitudinal distance matrices, showed significant correlations of *F*_*st*_ (*r* = 0.75, *t* = 2.48, *P*_*two-tailed*_ = 0.013) with geographic distance according to the hypothesis of isolation by distance. In addition, *F*_*st*_ (*r* = −0.65, *t* = −2.22, *P*_*two-tailed*_ = 0.025) correlated inversely with altitude. In contrast, the results from AMOVA indicated a lack of population structure, with a 94.95% of overall variation within populations (Table 1).

**Table 1:** Molecular analysis of variance (AMOVA) at Sierra de Gredos populations

Source	df	SS	Varcomp	% Var
Among groups	2	23.66	0.053	2.69
Among populations within groups	2	10.58	0.046	2.35
Within populations	369	693.90	1.880	94.96
Total	373	728.15	1.980	100

df, degree freedom; SS, sum of squares; Varcomp, variance components; % Var, proportion of total variance attributable to each source.

### Latitudinal genetic variation

**Figure 4:**
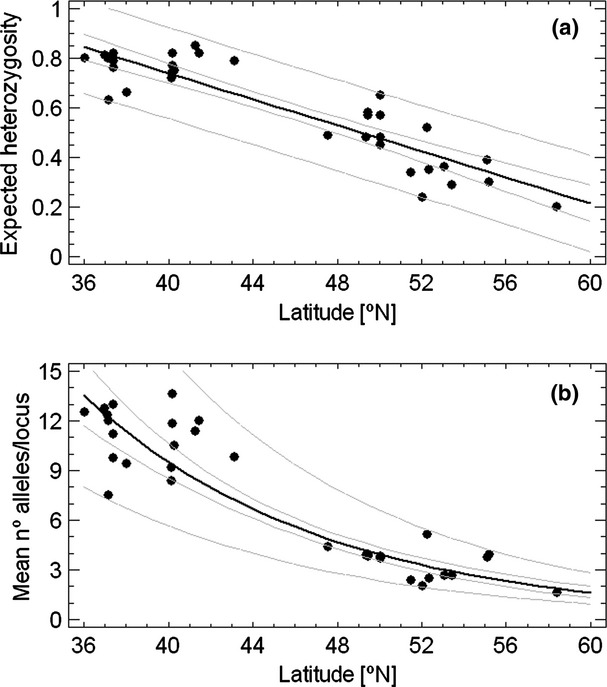
Genetic diversity and latitude relationship. Mean expected heterozygosity (a) and allelic richness (b) for each population. Each point represents a population of this study and compiled from literature (see Supporting Table S2 for details).

**Figure 5:**
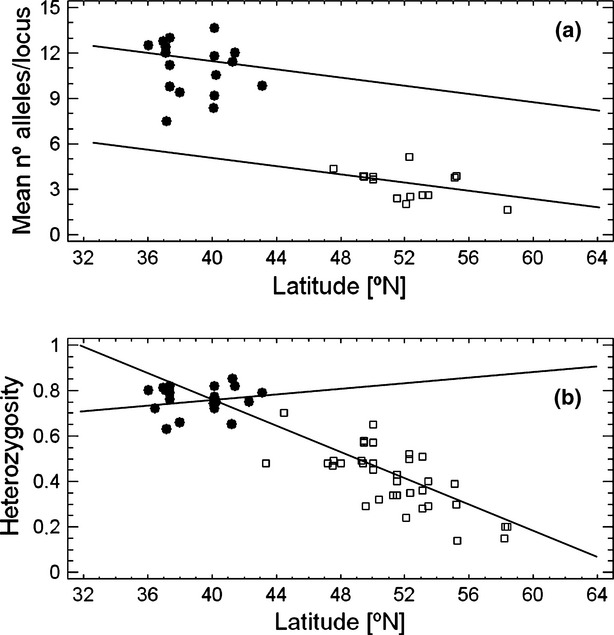
Genetic diversity and latitude relationship. The separately analysis considering the Iberian Peninsula as the glacial refuge. Mean expected heterozygosity (a) and mean number of alleles per locus (b). Each point (black circles, Iberian populations; white squares, northern populations) represents a population.

In the 57 European populations distributed in a south–north latitudinal gradient, *H*_*E*_ correlated inversely with latitude, explaining 80.46% of the variance in percentage (regression model: *H*_*E*_ = 1.834 − 0.028 × latitude; *r* = −0.90, *P* < 0.00001; Fig. 4a). *M*_*a*_ also showed an inversely significant relationship with latitude, explaining 81.35% of the variance (regression model: *M*_*a*_ = exp [5.789 − 0.089 × latitude]; *r* = −0.928, *P* < 0.00001; Fig. 4b). The separate analysis considering the Iberian Peninsula as the glacial refuge showed that *H*_*E*_ and *M*_*a*_ did not vary significantly with the latitude in the Iberian populations (*H*_*E*_*,* lineal regression model: *r* = 0.02, *P* = 0.92, Fig. 5a; *M*_*a*_ exponential regression model: *r* = −0.16, *P* = 0.53, Fig. 5b), whereas these parameters correlated inversely across the latitudinal gradient due to the barrier of the Pyrenees to the north (*H*_*E*_ regression model: *H*_*E*_ = exp [3.329 − 0.083 × latitude], *r* = −0.74, *P* < 0.0001, *R*^2^ = 52.74%, Fig. 5a; *M*_*a*_ regression model: *M*_*a*_ = exp [4.476 − 0.064 × latitude], *r* = −0.56, *P* = 0.017, *R*^2^ = 32.24%, Fig. 5b). Our results emphasize a decrease in genetic diversity at the glacial barrier to north latitudinal gradient in the European *B. calamita* distribution and a wide variation in genetic diversity in the Iberian Peninsula.

## Discussion

Amphibians are considered particularly vulnerable to environmental changes as a consequence of their low capacity for dispersal (Blaustein et al. 1994). Genetic studies in amphibians have been mainly focused in locally distributed populations with a high risk of isolation, especially those living in fragmented habitats, which can be at risk of extinction from demographic, environmental, and genetic stochasticity (reviewed in Allentoft and O'Brien 2010). Nevertheless, the effects of environmental factors such as altitude and latitude on genetic differentiation can only be addressed by analyzing data from species with a wide geographic range of distribution, as is the case of *B. calamita*. Our study is the first to analyze the genetic variation in *B. calamita* across altitudinal ranges. First we demonstrated how altitude constitutes a barrier decreasing genetic diversity from low altitudes to high altitudes, and second how the Pyrenees constituted a natural barrier across an European latitudinal gradient, demonstrating the relevance of mountain systems as barriers for amphibian populations.

### Altitudinal variation and population genetic diversity

Our results show that altitude is inversely correlated with genetic diversity of *B. calamita* populations in the Sierra de Gredos. Geographic isolation at high altitudes and a low population size could explain these results. Although we are not able to estimate population size, a low population size in high altitudes of Sierra de Gredos can be assumed because we observe both: a low number of individuals during the activity period and a low number of spawns in the breeding sites. Despite the decrease in genetic diversity found in highland populations of the Sierra de Gredos, *H*_*E*_ and *M*_*a*_ values are close to those observed in lowland populations of the south Iberian Peninsula (Gomez-Mestre and Tejedo 2004; Marangoni 2006). Therefore, these results emphasized the high variability in genetic diversity in the Iberian Peninsula probably due to the landscape heterogeneity.

*F*_*st*_ and geographic distance showed significant correlations according to the isolation by distance pattern. A significant correlation was also found between *F*_*st*_ and altitude. We noted that, in addition to geographic distance, the altitude distance analyzed in our study included some intrinsic variables, such as temperature, slope, and forest cover. Therefore, we suggest that the levels of genetic differentiation among populations of *B. calamita* in the altitudinal range of Sierra de Gredos are due to the combined effects of discontinuous habitat, produced mainly by the high slopes barriers, and geographic distance. As Marangoni (2006) did not find differences between populations in a geographic distance of more than 100 km in a lowland area, we can consider the differences found in our study to be mainly contributed by the altitudinal gradient.

In despite of the significant variation in *F*_*st*_ in the geographic and altitudinal gradient, the low *F*_*st*_ – statistics and the results of AMOVA indicated that there has been significant gene flow between all populations over historical time (historical perspectives). The landscape features seem to be adequate for gene flow, influencing genetic variations within and between populations (Stéphanie et al. 2003). Therefore, the effects of altitude in the genetic population structure seem to be minor, as suggested by the high gene flow observed between populations in different altitudes which can induce an incomplete separation of populations over large geographic (Brede and Beebee 2004) and altitudinal (this study) distances. The correlations found between genetic distance and altitudinal and geographic distance are probably due to the habitat discontinuities (especially high slopes) that generate less interconnection among populations. In fact, connectivity of local populations by dispersers is great in the Iberian Peninsula indicating a considerably large metapopulation system (Sinsch et al. 2012). The differentiation among natterjack toad populations is only remarkable in fragmented habitats where populations are isolated (Allentoft et al. 2009; Rogell et al. 2010).

### Latitudinal genetic differentiation

Genetic diversity of Sierra de Gredos populations differs from that of other European regions. Following the genetic characteristics found in the Iberian Peninsula (Gomez-Mestre and Tejedo 2004; Marangoni 2006), Gredos populations had higher allelic diversity than the same species in Europe (Rowe et al. 1998; Beebee and Rowe 2000; Rowe et al. 2006). Whereas in Gredos populations the *M*_*a*_ ranged between 8.38 and 13.63, populations in northern Europe ranged between 1.63 and 5.13 (Beebee and Rowe 2000; Frantz et al. 2009). It was notable that genetic diversity in the extremes of species distribution is low, for example, *M*_*a*_ in Poland was 2, despite the fact that populations in these areas are numerous and large (Beebee and Rowe 2000). Populations are more isolated in the peripheral regions of the *B. calamita* distribution area (Beebee 1983; Rogell et al. 2010) and the genetic variation decreased as a result of increased population differentiation (Petit et al. 2001). These results have been supported in studies about migratory ranges carried out in populations from Britain, which considered that these populations cannot maintain connectivity of neighboring local populations (Sinsch et al. 2012). On the contrary, metapopulation dynamics seem to be much greater in populations from the Iberian Peninsula (Sinsch et al. 2012) with high genetic diversity and gene flow (Marangoni 2006).

As we noted, the *M*_*a*_ and *H*_*E*_ variables showed a decrease in genetic diversity in the latitudinal gradient of *B. calamita* distribution. This difference is especially exemplified with *M*_*a*_, which shows a relatively high range of variation in the populations from Spain without latitudinal influence. On the contrary, in the populations from north of the Pyrenees to Sweden (at 49–58.5ºN) genetic variability decreases in the latitudinal gradient. These results are consistent with the hypothesis that the Iberian Peninsula was a refuge for *B. calamita* during the Pleistocene, where all populations expanded from the south to the north during the postglacial period (Beebee and Rowe 2000; Rowe et al. 2006). However, the incorporation of our data, in which the wide latitudinal variation in the Iberian Peninsula is included, contributes to a better interpretation of the colonization event. In the previous studies, Beebee and Rowe (2000) and also Rowe et al. (2006) have found that *H*_*E*_ correlated strongly with the geographic distance measured from south Spain. Our new data show that this correlation is not significant in the Iberian Peninsula that present a high genetic diversity; although it is strongly notable from the barrier of the Pyrenees to the north.

In conclusion, the pattern of genetic diversity of *B. calamita* showed throughout its geographic distribution area mainly reflects the colonization history of the species after the last glacial period. This was suggested by previous studies in the latitudinal gradient (Beebee and Rowe 2000; Rowe et al. 2006) and by this study at both latitudinal and altitudinal gradients. However, in our study we emphasize that the latitudinal variation is only notable since the barrier of the Pyrenees to the north. In addition, the studies of genetic structure (this study) and home range (Sinsch et al. 2012) evidenced that some populations can be genetically isolated by distance and prone to local extinction.

## Supporting Information

**Table S1.** Population description.

**Table S2.** Genetic diversity parameters of populations included in the study.

**Table S3.** Genetic diversity parameters at Sierra de Gredos sampled sites.
