# Supplementary material for: Genetic variability in geographic populations of the natterjack toad (Bufo calamita)
Source: Ecol Evol. 2012 Jul 20;2(8):2018–26. doi: 10.1002/ece3.323 (PMC3434004; doi:10.1002/ece3.323)
Supplement: Supplementary file 1 [file ece30002-2018-SD1.docx]

**APPENDIX**

**Table 1.** Population description.

| **Population** | **Coordinates** | **Altitude** [m] | **Date of collection** | **male/female** |
| --- | --- | --- | --- | --- |
| Navaluenga | 4024'57.33''N 440'47.45''W | 752 | April 2008 | 32/21 |
| Dehesa del Barraco | 4027'44,53"N 438'12,37"W | 920 | March 2010 | 39/11 |
| La Cedrera | 4030'33,52"N 439'16,41"W | 1472 | March 2010 | 35/16 |
| Cavadores | 4016'19.61''N 515'12.94''W | 2100 | May 2009 | 31/1 |
| Navasomera | 4015'07.31''N 515'49.47''W | 2270 | June 2008 | 36/5 |

AAP Annual Activity Period

**Table 2.** Genetic diversity parameters of populations included in the study.

| **Populations** | **Microsatellites used** | | | | | | | | | | | | | ***Diversity parameters*** | | | **Geographical parameters** | | **References** |
| --- | --- | --- | --- | --- | --- | --- | --- | --- | --- | --- | --- | --- | --- | --- | --- | --- | --- | --- | --- |
|  | Bcalμ1 | Bcalμ2 | Bcalμ3 | Bcalμ4 | Bcalμ5 | Bcalμ6 | Bcalμ7 | Bcalμ8 | Bcalμ9 | Bcalμ10 | Bcalμ11 | Buca2 | Buca6 | *M_a_* | *H_E_* | *H_o_* | Latitude  [N] | Altitude  [m] |  |
| Abalario (Spain) | ✓ | ✓ | ✓ | ✓ | ✓ | ✓ | ✓ |  |  | ✓ | ✓ |  |  | 12.5 | 0.8 | 0.62 | 36.06 | 63 | Gómez-Mestre & Tejedo 2004 |
| Almeria(Spain) | ✓ | ✓ | ✓ | ✓ | ✓ | ✓ | ✓ | ✓ |  |  |  |  |  |  | 0.72 |  | 36.5 | 70 | Rowe *et al*. 2006 |
| RBD (Spain) | ✓ | ✓ | ✓ | ✓ | ✓ | ✓ | ✓ |  |  | ✓ | ✓ |  |  | 12.75 | 0.81 | 0.62 | 37 | 24 | Marangoni 2006 |
| Bodegones (Spain) | ✓ | ✓ | ✓ | ✓ | ✓ | ✓ | ✓ |  |  | ✓ | ✓ |  |  | 12.37 | 0.8 | 0.52 | 37.13 | 32 | Marangoni 2006 |
| Juncosilla (Spain) | ✓ | ✓ | ✓ | ✓ | ✓ | ✓ | ✓ |  |  | ✓ | ✓ |  |  | 12 | 0.8 | 0.61 | 37.17 | 20 | Marangoni 2006 |
| Toba (Spain) | ✓ | ✓ | ✓ | ✓ | ✓ | ✓ | ✓ |  |  | ✓ | ✓ |  |  | 7.5 | 0.63 | 0.53 | 37.19 | 950 | Gómez-Mestre & Tejedo 2004 |
| Sevilla (Spain) | ✓ | ✓ | ✓ | ✓ | ✓ | ✓ | ✓ | ✓ |  |  |  |  |  |  | 0.8 |  | 37.23 | 10 | Rowe *et al*. 2006 |
| Navas (Spain) | ✓ | ✓ | ✓ | ✓ | ✓ | ✓ | ✓ |  |  | ✓ | ✓ |  |  | 13 | 0.79 | 0.54 | 37.4 | 420 | Marangoni 2006 |
| Pedreso (Spain) | ✓ | ✓ | ✓ | ✓ | ✓ | ✓ | ✓ |  |  | ✓ | ✓ |  |  | 11.2 | 0.82 | 0.59 | 37.4 | 395 | Marangoni 2006 |
| Sanlúcar (Spain) | ✓ | ✓ | ✓ | ✓ | ✓ | ✓ | ✓ |  |  | ✓ | ✓ |  |  | 9.75 | 0.76 | 0.47 | 37.4 | 34 | Marangoni 2006 |
| Taraje (Spain) | ✓ | ✓ | ✓ | ✓ | ✓ | ✓ | ✓ |  |  | ✓ | ✓ |  |  | 9.38 | 0.66 | 0.63 | 38.04 | 420 | Gómez-Mestre & Tejedo 2004 |
| Navasomera (Spain) | ✓ | ✓ | ✓ | ✓ | ✓ | ✓ | ✓ |  |  | ✓ | ✓ |  |  | 8.37 | 0.74 | 0.69 | 40.15 | 2270 | This study |
| Cavadores (Spain) | ✓ | ✓ | ✓ | ✓ | ✓ | ✓ | ✓ |  |  | ✓ | ✓ |  |  | 9.15 | 0.72 | 0.67 | 40.16 | 2100 | This study |
| La Dehesa del Barraco (Spain) | ✓ | ✓ | ✓ | ✓ | ✓ | ✓ | ✓ |  |  | ✓ | ✓ |  |  | 11.8 | 0.77 | 0.75 | 40.2 | 920 | This study |
| Navaluenga (Spain) | ✓ | ✓ | ✓ | ✓ | ✓ | ✓ | ✓ |  |  | ✓ | ✓ |  |  | 13.62 | 0.82 | 0.82 | 40.2 | 750 | This study |
| La Cedrera (Spain) | ✓ | ✓ | ✓ | ✓ | ✓ | ✓ | ✓ |  |  | ✓ | ✓ |  |  | 10.5 | 0.75 | 0.79 | 40.3 | 1470 | This study |
| Barcelona (Spain) | ✓ | ✓ | ✓ | ✓ | ✓ | ✓ | ✓ | ✓ |  |  |  |  |  |  | 0.65 |  | 41.23 | 20 | Rowe *et al*. 2006 |
| Mas de Melons (Spain) | ✓ | ✓ | ✓ | ✓ | ✓ | ✓ | ✓ |  |  | ✓ | ✓ |  |  | 11.38 | 0.85 | 0.81 | 41.3 | 240 | Oromí, Fibla & Sanuy unpublished |
| Preixens (Spain) | ✓ | ✓ | ✓ | ✓ | ✓ | ✓ | ✓ |  |  | ✓ | ✓ |  |  | 12 | 0.82 | 0.82 | 41.47 | 326 | Oromí, Fibla & Sanuy unpublished |
| Leon (Spain) | ✓ | ✓ | ✓ | ✓ | ✓ | ✓ | ✓ | ✓ |  |  |  |  |  |  | 0.75 |  | 42.35 | 830 | Rowe *et al*. 2006 |
| Batchouta (Spain) | ✓ | ✓ | ✓ | ✓ | ✓ | ✓ | ✓ |  |  | ✓ | ✓ |  |  | 9.8 | 0.79 | 0.7 | 43.14 | 700 | Oromí, Nicieza & Tejedo unpublished |
| Carmargue (France) | ✓ | ✓ | ✓ | ✓ | ✓ | ✓ | ✓ | ✓ |  |  |  |  |  |  | 0.48 |  | 43.35 | 20 | Rowe *et al*. 2006 |
| Bordeaux (France) | ✓ | ✓ | ✓ | ✓ | ✓ | ✓ | ✓ | ✓ |  |  |  |  |  |  | 0.7 |  | 44.5 | 20 | Rowe *et al*. 2006 |
| Zurich (Switzerland) | ✓ | ✓ | ✓ | ✓ | ✓ | ✓ | ✓ | ✓ |  |  |  |  |  |  | 0.48 |  | 47.22 | 430 | Rowe *et al*. 2006 |
| South Brittany (France) | ✓ | ✓ | ✓ | ✓ | ✓ | ✓ | ✓ | ✓ |  |  |  |  |  |  | 0.47 |  | 47.5 | 0 | Rowe *et al*. 2006 |
| Brittany (France) | ✓ | ✓ | ✓ | ✓ | ✓ | ✓ | ✓ | ✓ |  |  |  |  |  | 4.38 | 0.49 | 0.35 | 47.57 | 0 | Beebee & Rowe 2000 |
| West Brittany (France) | ✓ | ✓ | ✓ | ✓ | ✓ | ✓ | ✓ | ✓ |  |  |  |  |  |  | 0.48 |  | 48 | 0 | Rowe *et al*. 2006 |
| Cherbourg (France) | ✓ | ✓ | ✓ | ✓ | ✓ | ✓ | ✓ | ✓ |  |  |  |  |  |  | 0.49 |  | 49.29 | 0 | Rowe *et al*. 2006 |
| Boulogne (France) | ✓ | ✓ | ✓ | ✓ | ✓ | ✓ | ✓ | ✓ |  |  |  |  |  | 3.88 | 0.48 | 0.45 | 49.39 | 0 | Beebee & Rowe 2000 |
| Ernzen (Luxemburg) | ✓ |  | ✓ | ✓ | ✓ | ✓ |  | ✓ | ✓ |  | ✓ | ✓ | ✓ | 3.9 | 0.58 | 0.63 | 49.48 | 300 | Frantz *et al*. 2009 |
| Steinfort (Luxemburg) | ✓ |  | ✓ | ✓ | ✓ | ✓ |  | ✓ | ✓ |  | ✓ | ✓ | ✓ | 3.8 | 0.57 | 0.58 | 49.48 | 300 | Frantz *et al*. 2009 |
| Bieloweiza (Poland) | ✓ | ✓ | ✓ | ✓ | ✓ | ✓ | ✓ | ✓ |  |  |  |  |  |  | 0.29 |  | 49.56 | 0 | Rowe *et al*. 2006 |
| FLC (Belgium) | ✓ | ✓ |  |  | ✓ | ✓ | ✓ |  |  | ✓ |  |  |  | 3.67 | 0.65 | 0.65 | 50.05 | 160 | Stevens *et al*. 2006 |
| MAR (Belgium) | ✓ | ✓ |  |  | ✓ | ✓ | ✓ |  |  | ✓ |  |  |  | 3.83 | 0.45 | 0.45 | 50.05 | 150 | Stevens *et al*. 2006 |
| MER (Belgium) | ✓ | ✓ |  |  | ✓ | ✓ | ✓ |  |  | ✓ |  |  |  | 3.67 | 0.48 | 0.48 | 50.05 | 220 | Stevens *et al*. 2006 |
| ROM (Belgium) | ✓ | ✓ |  |  | ✓ | ✓ | ✓ |  |  | ✓ |  |  |  | 3.83 | 0.57 | 0.57 | 50.05 | 180 | Stevens *et al*. 2006 |
| Boulogne (France) | ✓ | ✓ | ✓ | ✓ | ✓ | ✓ | ✓ | ✓ |  |  |  |  |  |  | 0.32 |  | 50.36 | 0 | Rowe *et al*. 2006 |
| Halle (Germany) | ✓ | ✓ | ✓ | ✓ | ✓ | ✓ | ✓ | ✓ |  |  |  |  |  |  | 0.34 |  | 51.28 | 400 | Rowe *et al*. 2006 |
| Nijmegen (Netherlands) | ✓ | ✓ | ✓ | ✓ | ✓ | ✓ | ✓ | ✓ |  |  |  |  |  |  | 0.43 |  | 51.5 | 150 | Rowe *et al*. 2006 |
| Kerry (Ireland) | ✓ | ✓ | ✓ | ✓ | ✓ | ✓ | ✓ | ✓ |  |  |  |  |  | 2.38 | 0.34 | 0.33 | 51.52 | 0 | Beebee & Rowe 2000 |
| Kerry (Ireland) | ✓ | ✓ | ✓ | ✓ | ✓ | ✓ | ✓ | ✓ |  |  |  |  |  |  | 0.4 |  | 51.52 | 0 | Rowe *et al*. 2006 |
| Poland | ✓ | ✓ | ✓ | ✓ | ✓ | ✓ | ✓ | ✓ |  |  |  |  |  | 2 | 0.24 | 0.28 | 52.06 | 0 | Beebee & Rowe 2000 |
| East Anglia (England) | ✓ | ✓ | ✓ | ✓ | ✓ | ✓ | ✓ | ✓ |  |  |  |  |  |  | 0.5 |  | 52.29 | 0 | Rowe *et al*. 2006 |
| Ooy-Polder (Holland) | ✓ | ✓ | ✓ | ✓ | ✓ | ✓ | ✓ | ✓ |  |  |  |  |  | 5.13 | 0.52 | 0.46 | 52.29 | 0 | Beebee & Rowe 2000 |
| E/SE (England) | ✓ | ✓ | ✓ | ✓ | ✓ | ✓ | ✓ | ✓ |  |  |  |  |  | 2.5 | 0.35 | 0.3 | 52.35 | 0 | Beebee & Rowe 2000 |
| Texel (Holland) | ✓ | ✓ | ✓ | ✓ | ✓ | ✓ | ✓ | ✓ |  |  |  |  |  | 2.63 | 0.36 | 0.43 | 53.08 | 0 | Beebee & Rowe 2000 |
| Texel (Netherlands) | ✓ | ✓ | ✓ | ✓ | ✓ | ✓ | ✓ | ✓ |  |  |  |  |  |  | 0.28 |  | 53.08 | 150 | Rowe *et al*. 2006 |
| Duddon (England) | ✓ | ✓ | ✓ | ✓ | ✓ | ✓ | ✓ | ✓ |  |  |  |  |  |  | 0.51 |  | 53.1 | 0 | Rowe *et al*. 2006 |
| Merseyside (England) | ✓ | ✓ | ✓ | ✓ | ✓ | ✓ | ✓ | ✓ |  |  |  |  |  | 2.63 | 0.29 | 0.29 | 53.44 | 0 | Beebee & Rowe 2000 |
| Merseyside (England) | ✓ | ✓ | ✓ | ✓ | ✓ | ✓ | ✓ | ✓ |  |  |  |  |  |  | 0.4 |  | 53.44 | 0 | Rowe *et al*. 2006 |
| Cumbria (England) | ✓ | ✓ | ✓ | ✓ | ✓ | ✓ | ✓ | ✓ |  |  |  |  |  | 3.75 | 0.39 | 0.34 | 55.1 | 0 | Beebee & Rowe 2000 |
| Denmark | ✓ | ✓ | ✓ | ✓ | ✓ | ✓ | ✓ | ✓ |  |  |  |  |  | 3.9 | 0.3 |  | 55.22 | 0 | Beebee & Rowe 2000 |
| Zealand (Denmark) | ✓ | ✓ | ✓ | ✓ | ✓ | ✓ | ✓ | ✓ |  |  |  |  |  |  | 0.14 |  | 55.27 | 0 | Rowe *et al*. 2006 |
| Uddevalla (Sweden) | ✓ | ✓ | ✓ | ✓ | ✓ | ✓ | ✓ | ✓ |  |  |  |  |  |  | 0.15 |  | 58.2 | 0 | Rowe *et al*. 2006 |
| Parnu (Estonia) | ✓ | ✓ | ✓ | ✓ | ✓ | ✓ | ✓ | ✓ |  |  |  |  |  |  | 0.2 |  | 58.23 | 0 | Rowe *et al*. 2006 |
| Sweden | ✓ | ✓ | ✓ | ✓ | ✓ | ✓ | ✓ | ✓ |  |  |  |  |  | 1.63 | 0.2 | 0.14 | 58.41 | 293 | Beebee & Rowe 2000 |

(*Ma)* Mean number of alelles per locus, expected (*H_E_*) and observed (*H_o_*) heterozygosity.

**Table 3.** Genetic diversity parameters at Sierra de Gredos sampled sites.

|  | **Nal750** | | | **Deh920** | | | **LaC1470** | | | **Cav2100** | | | **Nas2300** | | |
| --- | --- | --- | --- | --- | --- | --- | --- | --- | --- | --- | --- | --- | --- | --- | --- |
| **Locus** | **NA**(#e/#c) | **H_E_** | **H_o_** | **NA**(#e/#c) | **H_E_** | **H_o_** | **NA**(#e/#c) | **H_E_** | **H_o_** | **NA**(#e/#c) | **H_E_** | **H_o_** | **NA**(#e/#c) | **H_E_** | **H_o_** |
| ***Bcalμ1*** | 17 (4/13) | 0.93 | 0.85 | 13 (1/12) | 0.84 | 0.65 | 10 (0/10) | 0.75 | 0.58 | 11 (1/10) | 0.80 | 0.56 | 12 (0/12) | 0.89 | 0.69 |
| ***Bcalμ2*** | 12 (2/10) | 0.82 | 0.74 | 12 (3/9) | 0.80 | 0.49 | 13 (2/11) | 0.72 | 0.76 | 6 (1/5) | 0.45 | 0.28 | 2 (0/2) | 0.51 | 0.29 |
| ***Bcalμ3*** | 12 (4/8) | 0.70 | 0.72 | 7 (2/5) | 0.66 | 0.78 | 9 (2/7) | 0.76 | 0.75 | 7 (1/6) | 0.64 | 0.78 | 5 (0/5) | 0.58 | 0.60 |
| ***Bcalμ4*** | 18 (2/16) | 0.91 | 0.87 | 21 (4/17) | 0.89 | 0.98 | 16 (3/13) | 0.86 | 0.92 | 15 (1/14) | 0.91 | 0.90 | 16 (6/10) | 0.90 | 0.74 |
| ***Bcalμ5*** | 13 (3/10) | 0.82 | 0.85 | 12 (3/9) | 0.75 | 0.85 | 11 (2/9) | 0.79 | 0.93 | 10 (1/9) | 0.76 | 0.52 | 10 (2/10) | 0.79 | 0.78 |
| ***Bcalμ6*** | 18 (3/15) | 0.94 | 0.83 | 12 (1/11) | 0.86 | 0.66 | 12 (2/10) | 0.76 | 0.68 | 17 (1/16) | 0.92 | 0.87 | 15 (1/14) | 0.93 | 0.84 |
| ***Bcalμ7*** | 8 (2/6) | 0.67 | 0.77 | 10 (3/7) | 0.57 | 0.67 | 7 (1/6) | 0.61 | 0.88 | 4 (1/3) | 0.55 | 0.71 | 3 (0/3) | 0.62 | 0.89 |
| ***Bcalμ10*** | 11 (3/9) | 0.81 | 0.92 | 8 (2/6) | 0.79 | 0.98 | 6 (0/6) | 0.73 | 0.90 | 8 (2/6) | 0.77 | 0.72 | 4 (0/4) | 0.70 | 0.69 |
| **Total** | 109 |  |  | 175 |  |  | 95 |  |  | 78 |  |  | 67 |  |  |

NA, number of alleles; #e, number of specific alleles; #c, number of common alleles; *H_E_*, expected heterozygosity; *H_o_*, observed heterozygosity.
